# Role of acquired von Willebrand syndrome in the development of bleeding complications in patients treated with Impella RP devices

**DOI:** 10.1038/s41598-021-02833-8

**Published:** 2021-12-09

**Authors:** Mehmet Oezkur, Sara Reda, Heiko Rühl, Nils Theuerkauf, Stefan Kreyer, Georg Daniel Duerr, Efstratios Charitos, Miriam Silaschi, Marta Medina, Sebastian Zimmer, Christian Putensen, Hendrik Treede

**Affiliations:** 1 Department of Cardiovascular Surgery, University Hospital of Bonn, Bonn, Germany; 2 Department of Haematology, University Hospital of Bonn, Bonn, Germany; 3 Department of Anesthesiology and Intensive Care Medicine, University Hospital of Bonn, Bonn, Germany; 4 Department of Cardiology, University Hospital of Bonn, Bonn, Germany; 5 Department of Cardiovascular Surgery, University Hospital Mainz, Langenbeckstrasse 1, 55131 Mainz, Germany

**Keywords:** Cardiac device therapy, Risk factors, Heart failure

## Abstract

Axial flow pumps are standard treatment in cases of cardiogenic shock and high-risk interventions in cardiology and cardiac surgery, although the optimal anticoagulation strategy remains unclear. We evaluated whether laboratory findings could predict bleeding complications and acquired von Willebrand syndrome (avWS) among patients who were treated using axial flow pumps. We retrospectively evaluated 60 consecutive patients who received Impella devices (Impella RP: n = 20, Impella CP/5.0: n = 40; Abiomed Inc., Danvers, USA) between January 2019 and December 2020. Thirty-two patients (53.3%) experienced major or fatal bleeding complications (Bleeding Academic Research Consortium score of > 3) despite intravenous heparin being used to maintain normal activated partial thromboplastin times (40–50 s). Extensive testing was performed for 28 patients with bleeding complications (87.5%). Relative to patients with left ventricular support, patients with right ventricular support were less likely to develop avWS (87.5% vs. 58.8%, p = 0.035). Bleeding was significantly associated with avWS (odds ratio [OR]: 20.8, 95% confidence interval [CI]: 3.3–128.5; p = 0.001) and treatment duration (OR: 1.3, 95% CI 1.09–1.55; p = 0.003). Patients with avWS had longer Impella treatment than patients without avWS (2 days [1–4.7 days] vs. 7.3 days [3.2–13.0 days]). Bleeding complications during Impella support were associated with avWS in our cohort, while aPTT monitoring was not sufficient to prevent bleeding complications. A more targeted anticoagulation monitoring might be needed for patients who receive Impella devices.

## Introduction

Treatment of cardiogenic shock remains a clinical challenge despite recent innovations in the field of mechanical circulatory support (MCS). Impella devices (Abiomed Inc., Danvers, MA) were recently added to the MCS arsenal, alongside extracorporeal membrane oxygenation (ECMO), intra-aortic balloon pump, and the TandemHeart. Some studies have identified strategies for successfully treating cardiogenic shock, especially the combination of an Impella device with ECMO (“ECPELLA”)^1–5^. Although other contemporary studies have indicated that use of the Impella 2.5/CP devices is associated with adverse events and potentially higher mortality rates^6,7^. Data regarding vascular and bleeding complications in patients with Impella 5.0/5.5/RP or the combination therapy of Impella and ECMO are missing. Life-threatening severe bleeding may affect the ability to deliver further successful treatment^6,7^. Peripheral bleeding, including access site bleeding, has also been discussed as a possible cause of increased mortality. However, there are no prospective studies that have specifically considered these complications.

Bleeding complications in patients who receive permanent or temporary MCS devices are associated with a risk of acquired von Willebrand syndrome (avWS)^8–15^. The von Willebrand factor (vWF) is a multimeric protein and the highest molecular weight multimers play a major role in primary hemostasis by binding to clot-associated collagen and platelet glycoprotein receptors, which helps seal the injured vascular endothelium. Furthermore, vWF is stored in the Weibel-Palade bodies of the endothelium as well as in platelet alpha granules. Therefore, vWF can be considered a plasma indicator of endothelial dysfunction and increased vascular vulnerability. Large vWF multimers are cleaved by the ADAMTS13 metalloprotease, especially under high shear stress conditions that promote the development of avWS^16^. Impaired hemostasis related to vWF abnormalities may also be a risk factor for procedural bleeding.

A type of avWS was first described in patients who had received axial continuous flow pumps and this syndrome was reversed after device explantation^17–19^. Federici et al. suggested that avWS could be identified based on a history of bleeding symptoms plus reduced vWF activity (< 50–65 IU/dL depending on blood type) and a reduced vWF activity-to-antigen ratio (< 0.7)^20,21^. Other researchers have also reported that a vWFn activity-to-antigen ratio of > 0.8, in the normal range, and lower values to be indicative of vWF dysfunction^22^.

However, critical illness and surgery can lead to a hypercoagulative state that is characterized by activation of coagulation and impairment of fibrinolysis. Thus, vWF activity and antigen levels are typically elevated in these settings, along with other coagulation factors, such as fibrinogen and coagulation factor VIII (FVIII), which can complicate the diagnosis of avWS. Moreover, there are limited data regarding the incidence of avWS and possible bleeding complications in patients who have received Impella RP devices. Therefore, this study evaluated patients who received left or right ventricular Impella support to determine whether the development of avWS was associated with bleeding complications. To the best of our knowledge, this is the largest single-center study to evaluate the relationship between avWS and bleeding complications in this setting.

## Methods

The study protocol and data handling of this retrospective cohort study was approved by the Ethics Committee of the Universityhospital of Bonn and the data protection officer. An informed consent for the retrospective analysis of the data was obtained from all subjects. All methods were performed in accordance with the relevant guidelines and regulations for good clinical and good scientific practice. We evaluated adult patients (≥ 18 years old) who had undergone elective or urgent cardiac surgery between January 2019 and December 2020 in the cardiac surgery at the University Hospital Bonn. These procedures included thoracic aorta procedures (n = 3) and coronary artery bypass grafting (CABG) (n = 36) with or without valve surgery (n = 41) (reconstruction or replacement). Patients were considered eligible if they had received an Impella CP/5.0/RP device at any point during their hospitalization. The exclusion criteria were removal of the Impella device and/or patient death during the first 24 h after implantation. All Impella RP devices had been implanted percutaneously via puncture of the femoral vein. All Impella 5.0 devices had been implanted surgically via the subclavian artery with a graft. All Impella CP devices had been implanted via puncture of the femoral artery. Explantation was performed via venous compression alone (Impella RP devices), using an 8-F Angio-Seal device (Impella CP devices), or surgically (Impella 5.0 devices).

All patients received a low dose i.v. heparin therapy before the implantation of the Impella devices aiming for an aPTT of 40–50 s as thrombosis prophylaxis. Patients who underwent CABG surgery additionally received 100 mg of acetylsalicylic acid. The ECMO sets as well as cannulas were heparin coated. The aPPT aim in patients with ECMO were 50–60 s.

The patients’ medical records were searched to collect data regarding patient characteristics, comorbidities, surgical procedures, hemodynamic parameters, laboratory findings, medications, and transfusion of blood products (coagulation factors, fresh-frozen plasma, and platelet concentrates). The data were collected at admission/preoperatively; at the time of Impella device implantation; at 24 h, 48 h, and 72 h after the implantation; and at 6 days after the implantation and/or at the explantation. The blood samples and the laboratory analyses were performed as part of the clinical routine. All patients had received intravenous heparin anticoagulation to maintain a partial thromboplastin time (PTT) of 50–60 s. PTT was tested twice per day. We started with extended coagulation testing including aVWS testing when bleeding occurred. Later we performed the extended testing daily, beginning with implantation of an axial pump. The primary endpoint was defined as the development of avWS, which was identified based on the ratio of vWF activity (vWF:Ac) to von Willebrand factor antigen (vWF:Ag), with a value of < 0.70 any time between the implantation and explantation indicating the development of avWS^22,23^. The secondary endpoint was defined as major or fatal bleeding complications (Bleeding Academic Research Consortium [BARC] score of > 3) during the treatment^8–15,24^. Postoperative medical treatments in the intensive care unit were also evaluated.

Bleeding patients were treated according to best practice, depending on the findings of the extended analyses; including substitution of vWF concentrates.

Blood samples were centrifuged within 4 h and plasma samples were either immediately analyzed or stored at − 40 °C until they were analyzed. Plasma concentrations of vWF:AG were measured using immunoturbidimetry (Innovance vWF:Ag assay; Siemens Healthcare Diagnostics, Eschborn, Germany). The vWF:Ac was determined using a vWF ristocetin cofactor assay (Innovance vWF:Ac assay; Siemens)^25^. In addition to vWF:Ag, and vWF:Ac, we determined the following hemostasis parameters using an automated coagulation analyser (BCS XP or Atellica Coag 360, Siemens) and standard reagents: aPTT (Actin FS, Siemens), thrombin clotting time (TCT, Dade Thrombin, Siemens), fibrinogen levels (Clauss method, Dade Thrombin), plasma levels of coagulation factors (F) II, V, VII, VIII, X (Actin FS or Dade Innovin, Siemens), XIII (Berichrom Factor XIII, Siemens), antithrombin activity (Berichrom Antithrombin III) and D-dimer (INNOVANCE D-Dimer) were determined using an automated coagulation analyser (BCS XP or Atellica Coag 360, Siemens) and standard reagents. Reference ranges were established by analysing blood samples obtained from at least 100 healthy blood donors. Laboratory reference ranges were as follows: blood type 0: 50–130% for vWF:Ag and 46–125% for vWF:Ac; non-0 blood type: 65–165% for vWF:Ag and 64–150% for vWF:Ac,0.25–35 s for aPTT, < 20.5 s for TCT, 180–355 mg/dL for fibrinogen, 70–125% for FII, 68–135% for FV, 70–165% for FVII, 75–250% for FVIII, 70–120 s for FX, 70–155% for FXIII, 85–120% for antithrombin, and ≤ 0.5 mg/L for D-dimer. Reference ranges were established using blood test results from ≥ 100 healthy blood donors. Internal and external quality assurance was performed according to the German quality assurance guidelines for medical laboratory examinations.

### Statistical analysis

Data were presented as number (percentage) or median (interquartile range). The characteristics and outcomes of the Impella groups were compared using the t test, Mann–Whitney U test, Kruskal–Wallis H test, χ^2^ test, or Fisher’s exact test, as appropriate. Univariate regression analysis was performed to identify factors that were associated with bleeding complications, although multivariable analysis was performed we did not perform an extended analyses based on the small sample size. Differences were considered statistically significant at two-sided p-values of ≤ 0.05. All analyses were performed using SPSS software (version 25; IBM Corp., Armonk, NY).

## Results

**Figure 1:**
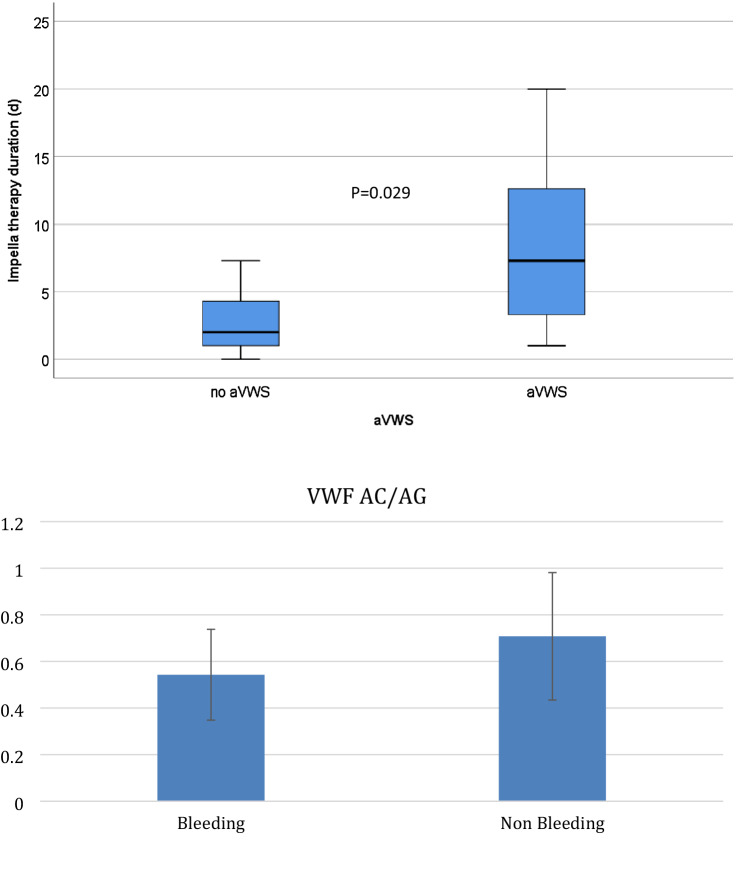
Impella treatment duration in patients who developed acquired von Willebrand syndrome. *avWS* acquired von Willebrand syndrome, *VWF* von Willebrand factor, *AC* von Willebrand activity, *AG* von Willebrand antigen.

During the study period, 60 patients received Impella RP devices (n = 20) or Impella CP/5.0 devices (n = 40) (Fig. 1, Table 1). Detailed coagulation analysis had been performed for 41 patients, although 1 patient was excluded because of device dislocation and an immediate switch to ECMO. Thirty-two patients (53.3%) patients experienced severe or major bleeding events (BARC score of ≥ 3) during treatment (Table 2). Of those 41 patients 28 had bleeding complications.. Additional ECMO was provided for 5 of 20 patients (25%) who received Impella RP devices and for 17 of 40 patients (42.3%) who received Impella CP/5.0 devices. There was no significant difference in bleeding complications between patients who did and did not receive ECMO (p = 0.49).

**Table 1:** Patient characteristics according to Impella use.

	All n = 60	LV Impella n = 40	RV Impella n = 20	p-value
Age, years	63 (54–70)	68.75 (62.0–75.7)	67.0 (56.25–76.75)	0.20
Impella duration, days	3.3 (1.15–7.85)	5.0 (1.15–9.4)	2.55 (1.12–7.22)	0.47
ECMO, n (%)	22 (36.7)	17 (42.5)	5 (22.5)	0.25
Major bleeding, n (%)	32 (53.3)	23 (57.5)	9 (45.0)	0.41
Access site bleeding, n (%)	1 (1,7%)	1 (2,5)	0 (0)	1.00
avWS, n (%)	31 (75.6)	21 (87.5)	10 (58.8)	0.035
Platelets, × 10^9^/L	117 (88–196)	130.0 (89.0–242.0)	110.0 (88.0–117.75)	0.33
Hb, g/dL	9.6 (8.7–10.9)	10.0 (9.0–11.8)	9.15 (8.52–9.6)	0.016
Fibrinogen, mg/dL	302.0 (224.25–394.5)	332.5 (242.25–505.0)	299.0 (187.5–340.75)	0.33
Factor II, %	63.0 (50.0–75.75)	62.5 (51.0–75.5)	65.0 (39.75–81.25)	0.99
Factor V, %	59.0 (45.5–75.5)	59.0 (48.0–75.5)	58.5 (32.75–81.75)	0.82
Factor VII, %	53.0 (34.0–71.0)	53.0 (35.0–61.0)	56.0 (31.5–83.0)	0.60
Factor VIII, %	356.9 (281.7–450.0)	356.55 (282.52–450.0)	450.0 (238.8–450.0)	0.62
Factor X, %	57.0 (47.5–79.0)	57.0 (49.0–72.5)	67.0 (35.5–95.0)	0.72
Factor XIII, %	78.0 (65.0–94.0)	83.0 (66.0–98.0)	76.5 (63.0–87.75)	0.37
AT III, %	63.0 (54.0–74.0)	63.5 (54.25–74.5)	62.0 (44.0–74.0)	0.42
γGT, U/L	45.5 (29.25–89.25)	70.5 (34.5–128.5)	39.5 (34.25–57.5)	0.051
ALAT, U/L	59.0 (26.0–166.0)	62.0 (27.25–166.0)	56.5 (25.0–171.0)	0.65
ASAT, U/L	131.5 (40.75–458.0)	142.0 (33.25522.5)	131.5 (54.25–352.0)	0.83
TCT s	10.3 (9.2–12.57)	10.8 (9.42–12.17)	9.9 (8.65–14.97)	0.36
aPTT	35.0 (26.0–45.0)	35.4 (26.0–44.0)	34.5 (26.5–48.75)	0.83
LDH U/l	616.0 (355.0–1472.0)	537.0 (324.5–1263.0)	1282.0 (433.75–3209.5)	0.081
RC n	8.5 (0–22.25)	12.0 (0–23)	5 (0–12)	0.24
PC n	3 (1–6)	2.5 (1–6.25)	5 (2–6)	0.43
FFP n	1 (1–2)	5 (0–14.5)	6 (0–15.0)	0.82

Data are shown as number (%) or median (interquartile range).

*ECMO* extra corporal membrane oxygenation, *avWS* acquired von Willebrand syndrome, *Hb* hemoglobin, *ATIII* antithrombin III, *γGT* γ-glutamyl transferase, *ALAT* alanine aminotransferase, *ASAT* aspartate aminotransferase, *TCT* thrombin clotting time, *aPTT* activated partial thromboplastin time, *LDH* lactate dehydrogenase, *RC* red cell concentrate, *PC* platelet concentrate, *FFP* fresh frozen plasma.

**Table 2:** Patient characteristics according to bleeding status.

	All	Bleeding (n = 32)	No bleeding (n = 28)	p-value
Age, years	63 (54–75)	66 (55.75–71)	58.5 (46.5–67.5)	0.096
Hb, g/dL	9.6 (8.7–10.9)	9.8 (9.0–11.47)	9.3 (8.5–10.9)	0.32
Platelets, × 10^9^/L	117.0 (88.0–196.0)	110 (90–182.25)	130 (88–242)	0.63
γGT, U/L	45.5 (29.25–89.25)	47.5 (34.5–95.25)	40.5 (21.25–87.25)	0.29
ALAT, U/L	59.0 (29.25–89.25)	69.0 (25.25–166.0)	58.5 (26.25–271.0)	0.79
ASAT, U/L	131.5 (40.75)	102.0 (32.25–388.0)	223.5 (55.25–585.75)	0.34
LDH, U/L	616.0 (355.0–1,472.0)	618 (331.0–2,382.0)	590 (363.25–1,321.25)	0.85
avWS, n (%)	31 (75.6)	26 (92.9)	5 (38.5)	< 0.001
Uncontrolled, n (%)	19 (31.7)	4 (12.5)	15 (53.6)	0.001
RC, n	8.5 (0–22.25)	17.0 (4.25–23.75)	2.5 (0–11.0)	0.005
PC, n	3 (1–6)	4 (2–8.5)	2 (0.5–5.0)	0.056
FFP, n	5.5 (0–14.5)	6 (3–20.5)	0 (0–10)	0.063
ECMO, n (%)	22 (36.7)	13 (40.6)	9 (32.1)	0.49
Access site bleeding, n (%)	1 (1.7)	1 (3.1)	0 (0)	1.0

Data are presented as number (%) or median (interquartile range).

*γGT* γ-glutamyl transferase, alanine aminotransferase; *ASAT* aspartate aminotransferase, *LDH* lactate dehydrogenase, *avWS* acquired von Willebrand syndrome, *Uncontrolled* patients missing extensive analyses, *RC* red cell concentrate, *PC* platelet concentrate, *FFP* fresh frozen plasma, *ECMO* extra corporal membrane oxygenation.

We identified avWS in 31 patients, which was associated with bleeding complications (BARC score of ≥ 3) (odds ratio [OR]: 20.8, 95% confidence interval [CI]: 3.36–128.53; p = 0.001). Patients with right-side Impella RP devices were significantly less likely to develop avWS, relative to patients who received left-side Impella CP/5.0 devices (10/17 patients [53.5%] vs. 21/24 patients [87.5%]; p = 0.035). Furthermore, bleeding complications were associated with each additional day of treatment (OR: 1.3, 95% CI 1.09–1.55; p = 0.003) (Tables 3). However, the PTT values were not associated with bleeding complications in our cohort. No access site bleeding was observed in the Impella RP group, although 1 patient (2.5%) in the Impella CP/5.0 group experienced access site bleeding. No thrombotic complications were observed in any group.

**Table 3:** Univariate regression analysis of bleeding risk.

	Odds ratio	95% CI	p-value
avWS	20.8	3.36–128.53	0.001
Impella duration	1.3	1.09–1.55	0.003
aPTT at onset of bleeding	1.0	0.98–1.02	0.98

Variable	Odds ratio	95% CI	p-value
avWS	14.78	2.14–101.94	0.006
Impella duration	1.07	0.89–1.28	0.44

*CI* confidence interval, *avWS* acquired von-Willebrand-Syndrome.

## Discussion

This study revealed that patients who received Impella RP devices were less likely to develop avWS during their treatment, relative to patients who received Impella 5.0/CP devices. Furthermore, the development of avWS was associated with bleeding complications in our cohort. Moreover, we did not identify any cases of thrombotic complications or access site bleeding in the Impella RP group. The combination of ECMO and Impella therpy was not associated with a higher incidence of bleeding complications nor with the development of avWS. Other coagulation analyses performed (factors II, V, VII, VIII, X, XIII, AT III, thrombin time, fibrogen and platlet count) were not associated with bleeding complications.

Cases of avWS are relatively common after MCS therapy, with reported incidences of 30–100% depending on the type of support, device, and measurement method^8,9,14,15^. Furthermore, avWS is associated with an increased risk of bleeding among patients who require MCS^8,9,13,18,19,26^. In patients with left ventricular assist devices, avWS is associated with increased risks of early and long-term bleeding complications^8,14^. In patients who require temporary MSC, such as ECMO, avWS is also very common and is associated with an increased risk of major bleeding complications^11,12,15^. The severity of the bleeding complications depends on comorbidities, the duration of MCS, and anticoagulation or platelet inhibition during MCS^27^.

This study revealed that the patients had elevated baseline values for vWF:Ag and vWF:Ac, with a normal activity-to-antigen ratio. Although none of the patients fulfilled the diagnostic criteria for possible avWS preoperatively, many exhibited reduced vWF function that was reflected in a reduced activity-to-antigen ratio. In this context, avWS is caused by the loss of large vWF multimers via increased vWF clearance, increased binding to cell surfaces, and/or proteolytic loss. During MCS, avWS is predominantly related to increased proteolytic cleavage of vWF by ADAMTS13, which is promoted by shear stress-dependent conformational changes in these multimers. This cleavage increases the risk of bleeding among patients who require MCS. In our cohort, despite similar pump power and support, avWS was less common in the group that received right ventricular support (vs. left ventricular support), which might be related to lower pressures and less shear stress at the right ventricular site. Another possible explanation is that the group that received left ventricular support was more likely to receive both ECMO and an Impella device, which would suggest that two MCS strategies could increase the risk of avWS. However, we failed to detect a significant difference in avWS or major bleeding complications according to the use or non-use of ECMO.

Bleeding complications after Impella CP/5.0 device implantation may compromise the outcomes in patients with cardiogenic shock^6,21^. In particular, access site complications have been discussed as potential causes of major bleeding. Unfortunately, previous studies have provided limited information regarding anticoagulation strategies, bleeding locations, and temporal relationships with Impella device implantation. Therefore, further studies are needed to address these issues.

The Impella RP device is a novel mechanical right ventricular support system. However, given the relatively uncommon nature of isolated acute right ventricular failure, there is limited experience with Impella RP devices and most centers have performed < 10 implantations. Thus, given their early locations on the learning curve, most centers have been unable to provide useful data regarding access site bleeding and major bleeding complications in cases with Impella RP usage. To the best of our knowledge, our experience with 20 patients who received Impella RP devices is the largest single-center cohort at this time. We did not detect any right ventricular support cases with access site complications, which may be related to venous access being safe, based on the lower pressure and less calcification. Given the short learning curve, safe implantation for right and left ventricular support seems possible. Thus, while access site management might contribute to bleeding complications, it did not play a major role in our cohort.

We still observed a significant number of major and fatal bleeding events in our cohort (32 patients, 53%). This may be related to the use of these devices as a last resort in patients who were failing previous treatment, especially early in our learning curve, which might suggests that the patients had severe cardiogenic shock with or without right ventricular failure. Furthermore, after the first implantations, we used PTT and activated clotting time to control anticoagulation, while laboratory testing for other coagulation factors, such as vWF, was only performed for patients with bleeding complications. However, after clinical observation of bleeding complications, we established a standard operating procedure that included testing for coagulation factors and avWS screening. We suggest that anticoagulant management during Impella treatment should be differentiated beyond simple testing for activated clotting time or PTT. We could not find other coagulations factors associated with bleeding.

The present study has various limitations that should be considered. First, the retrospective analysis of data regarding avWS and bleeding events inherently selects for patients who underwent related testing, although this was not routinely performed during the first few months after this novel treatment was established. Thus, the results might be influenced by selection bias and a prospective study is needed to confirm the incidence of avWS during Impella treatment and its association with bleeding complications. Second, the analysis of vWF parameters was limited to vWF:Ag and vWF:Ac, which precludes a commentary regarding vWF multimers and the potential influences of ADAMTS13 on vWF parameters. Furthermore, it can be difficult to evaluate the vWF activity-to-antigen ratio in a situation with highly elevated vWF parameters, although certain patients with avWS-associated disorders may only exhibit reduced concentrations of high molecular weight vWF multimers during laboratory testing. Third, a large subgroup of patients was receiving preprocedural antiplatelet and anticoagulation therapy, which suggests that alternative causes of bleeding complications are possible. Fourth, the shear stress induced by centrifugal pumps depends on the rotational speed, and pump designs with different flow patterns may have different effects on coagulation parameters. Further studies of these differences are needed to guide optimal anticoagulation management in this setting. The hemodynamic findings are missing in the study. Although they might be of interest those findings would have been out of the scope of this study.

Based on the association between aVWS and bleeding complication observes in this study, we updated our diagnostic strategy for all patients who require axial flow pump support to routinely include testing for vWF:Ac. Furthermore, all patients who require MCS are screened for avWS before implantation, as well as factor deficiencies and avWS on days 1, 3, and 5. In cases with major bleeding and avWS, we administer factor VIII and vWF (Haemate) to achieve a platelet count of > 100,000/mL and normalize the aPTT. In cases with avWS but no bleeding, we aim for lower PTT values (35–40 s) and a platelet count of > 70,000/mL. We plan to evaluate the possible benefits of this more differentiated anticoagulation and therapeutic strategy in a prospective study.
